# Moving Towards Effective and Efficient Implementation of Evidence-Informed Deliberative Processes for Health Benefit Package Design: A Response to Recent Commentaries

**DOI:** 10.34172/ijhpm.8647

**Published:** 2024-07-20

**Authors:** Wija Oortwijn, Maarten Jansen, Leon Bijlmakers, Gavin Surgey, Rob Baltussen

**Affiliations:** IQ Health Science Department, Radboud University Medical Center, Nijmegen, The Netherlands

 We welcome the interest of several colleagues^1-7^ in our practical guide on evidence-informed deliberative processes (EDPs) for health benefit package (HBP) design.^8^ In their commentaries, published in this journal during 2023, all the authors applaud the development of the guidance and put forth several arguments for improvements and areas for further research, organised around three main themes: (*i*) how to best achieve legitimacy in practice, (*ii*) institutionalization of EDPs, and (*iii*) monitoring and evaluation (M&E). As authors involved in the process of developing and applying the guidance in practice, we would like to reflect on these arguments and suggestions, and explicitly would like to emphasize the need to move towards implementing EDPs in the most effective and efficient way.

## Addressing Legitimacy in Practice

 DiStefano^5^ argues that best practices for implementing EDPs may differ depending on whether the approach is grounded in moral versus social values. He suggests studying in-depth the use of EDPs in practice, including the need to assess how different approaches to appraisal (eg, more quantitative versus qualitative) impact perceptions of the value of deliberation itself. Bond^2^ argues that the need for social learning among stakeholders may require increased resourcing and that this blurs the boundary between moral deliberation and political negotiation. Guzman^3^ suggests there could be added value in prioritizing key issues of legitimacy within HBP processes, and acknowledges the costs and risks associated with the use of EDPs. We already suggested to document more comprehensively the ways in which legitimacy is addressed in HBP design and decision criteria are chosen in practice.^8^ Gopinathan^6^ rightly noted that EDPs have contributed to considerable progress for HBP design but also that investments in EDPs should be efficiently deployed to enhance the pre-existing legislative, institutional and political framework to promote fair and legitimate healthcare decisions in a transparent manner.

## Institutionalization

 Sajadi et al^4^ emphasize that institutionalization of EDPs must be considered, otherwise EDPs might not be integrated into the formal health system or be impactful, resulting in a waste of time and resources. For the institutionalization of EDPs, they suggest four stages: (1) establishing a supportive legal framework, (2) designating governance and institutional structure, (3) stipulating the EDPs processes, and (4) individual and institutional capacity building. Culyer^1^ also mentions the need for capacity building to raise awareness of health technology assessment (HTA), the use of knowledge translation, and exchange and deliberation amongst policy makers, especially in low- and middle-income countries. In addition, Nagpal et al^7^ argue that countries with established advisory committees can provide practical insights for countries in earlier stages of establishing a systematic process for HBP design. According to them peer-to-peer learning among countries could be an effective approach to institutionalizing EDPs. We agree with these reflections. In our EDP guide we dedicate a chapter to understanding the context, which covers the institutional design, the policy context, current levels of HTA capacity, and how each of these elements can be assessed. Furthermore, we are involved in Support Utilisation of Sustainable and TAilored INnovative methods for HTA (SUSTAIN-HTA), a European initiative supporting HTA bodies to mobilise new HTA methods amongst their workforce, building excellence in knowledge and skills and supporting the development of HTA internationally: https://sustain-hta.org/.

## Monitoring and Evaluation

 Finally, Guzman^3^ calls for clearer direction how HTA bodies can measure and report on compliance in each step of the HBP process and the benefits of doing so. DiStefano^5^ adds the need to identify appropriate outcome measures allowing to assess whether meeting the conditions for EDPs has the desired effects on legitimacy. Bond^2^ further notes that basing recommendations on processes that have changed over time and have not been well-evaluated may lead to sub-optimal legitimacy. We already recommended the use of outcome measures for legitimacy as part of the HTAi-ISPOR taskforce report on deliberative processes, in which tools to support M&E are provided,^9^ as well as in our recent work on routine M&E of HBP revision in Iran (article forthcoming). Bond agrees with us that robust M&E of EDPs is needed and that this should be based on an explicit theory of change. However, he also correctly points out that this crucial element seems much neglected by HTA bodies.

## Conclusion

 Overall, the EDP guidance has been well-received and is applied successfully in different countries.^10-12^ However, we also noted the appetite for more guidance on how users can make appropriate trade-offs between the various desirable features of EDPs to ensure an efficient EDP design that achieves its intended impacts. We therefore suggest developing a “choosing wisely” approach to the implementation of each step of the EDP framework that is monitored and evaluated over time. For example, setting clear principles and criteria for health technologies to be evaluated will improve the efficiency and predictability of that process. Another example involves flexibility of advisory committees in cases where it is difficult to generate enough evidence: how do different countries deal with uncertainty in their decision-making processes? Eventually choosing wisely, as well as a robust M&E mechanism will help implementing EDPs in a more pragmatic, agile, and effective way, while being adaptable to each jurisdiction.

## Ethical issues

 Not applicable.

## Competing interests

 Authors declare that they have no competing interests.
